# A Semi-Quantitative Method to Denote Generic Physical Activity Phenotypes from Long-Term Accelerometer Data – The ATLAS Index

**DOI:** 10.1371/journal.pone.0063522

**Published:** 2013-05-08

**Authors:** Michael Marschollek

**Affiliations:** Hanover Medical School, Peter L. Reichertz Institute for Medical Informatics, Hanover, Germany; University of California, Irvine, United States of America

## Abstract

**Background:**

Physical activity is inversely correlated to morbidity and mortality risk. Large cohort studies use wearable accelerometer devices to measure physical activity objectively, providing data potentially relevant to identify different activity patterns and to correlate these to health-related outcome measures. A method to compute relevant characteristics of such data not only with regard to duration and intensity, but also to regularity of activity events, is necessary. The aims of this paper are to propose a new method – the ATLAS index (Activity Types from Long-term Accelerometric Sensor data) – to derive generic measures for distinguishing different characteristic activity phenotypes from accelerometer data, to propose a comprehensive graphical representation, and to conduct a proof-of-concept with long-term measurements from different devices and cohorts.

**Methods:**

The ATLAS index consists of the three dimensions regularity (reg), duration (dur) and intensity (int) of relevant activity events identified in long-term accelerometer data. It can be regarded as a 3D vector and represented in a 3D cube graph. 12 exemplary data sets of three different cohort studies with 99,467 minutes of data were chosen for concept validation.

**Results:**

Five archetypical activity types are proposed along with their dimensional characteristics (*insufficiently active*: low reg, int and dur; *busy bee*: low dur and int, high reg; *cardio-active*: medium reg, int and dur, *endurance athlete*: high reg, int and dur; and *weekend warrior*: high int and dur, low reg). The data sets are displayed in one common graph, indicating characteristic differences in activity patterns.

**Conclusion:**

The ATLAS index incorporates the relevant regularity dimension apart from the widely-used measures of duration and intensity. Along with the 3D representation, it allows to compare different activity types in cohort study populations, both visually and computationally using vector distance measures. Further research is necessary to validate the ATLAS index in order to find normative values and group centroids.

## Background

Physical activity is a fundamental expression of human behaviour and closely connected to a person's well-being. There is a huge body of evidence indicating that physical activity is inversely correlated to morbidity and mortality: a low activity level may support the development of several diseases, whereas frequent physical activity lowers disease risks [1], [2]. While this general correlation between activity and morbidity has been proven, it remains unclear which types of activity or activity patterns, respectively, effect positive health outcomes, which do not, and what exactly causes these outcomes.

While until a few years ago large-scale cohort studies were dependent on activity questionnaires with limited validity [3], wearable activity monitors are used in several recent studies, e.g. UK Biobank (http://www.ukbiobank.ac.uk/), National Health and Nutrition Examination Survey (NHANES, http://www.cdc.gov/nchs/nhanes.htm) or the German National Cohort pre-study (www.nationale-kohorte.de/). Most of these devices contain a triaxial accelerometer, measuring linear accelerations. These devices provide large amounts of data which can be used in different ways to assess parameters such as the number of steps, gait speed, estimates of active energy expenditure, balance parameters, etc. [4]. Patterns of these parameters can be used e.g. to estimate individual fall risk [5]. Furthermore, the data allow for assessing detailed activity patterns that are not only based on duration and intensity, but also provide exact information about the regularity of activities. Often the analysis of the data is performed using proprietary and undisclosed algorithms provided by the device manufacturer, so that results may not be repeatable or fully comprehensive. Thus far, there is no generic published method to describe activity patterns using the fundamental dimensions duration, intensity and regularity for data of different accelerometer-based activity monitors.

Considering the rising relevance of a generic means to analyze and classify objective physical activity measurement data obtained over extended periods in large epidemiologic cohorts, the aims of the research for this paper are.

- to propose a new method to derive generic measures suitable for distinguishing different characteristic activity types: the *ATLAS* index (Activity Types from Long-term Accelerometric Sensor data, aim #1).
- to present a new three-dimensional graphical representation method for easy and comprehensive assessment of physical activity types (aim #2), and.
- to conduct a proof-of-concept study with exemplary long-term measurements from different devices and different cohort data sets (aim #3).

The remainder of this paper is organized as follows: The sub-section ‘related work’ briefly covers research related to the definitions of physical activity patterns and activity recommendations as well as to the effects of known patterns such as the ‘weekend warrior’. The ‘methods’ section reports on the approach to derive the ATLAS score from accelerometer data, on the proposed graphical representation of score results and on the data sets with which the proof-of-concept was performed. In the discussion chapter, the results are related to research work of other groups and discussed in the light of obvious application areas of the new method. The conclusion summarizes the main findings.

### Related work

The American Heart Association (AHA) and the American College of Sports Medicine (ACSM) recommend an activity pattern of 5 times per week on separate days with each 30 minutes duration with moderate intensity (3 to 6 metabolic units, METs) or 3 times with 20 minutes of vigorous activity (>6 METs) [6]. While numbers are equal for older adults, a different recommendation is given for the intensity of activities: Instead of the above-mentioned MET levels, which are difficult to reach by elderly persons with low fitness, the authors recommend to use target percentage values of individual maximum oxygen uptake [7]. The World Health Organization has recently published physical activity recommendations: at least 150 minutes of moderate or 75 minutes of vigorous aerobic activity per week for adults, each with a minimum duration of ten minutes [8]. For an optimal health-related outcome, 300 (moderate) resp. 150 (vigorous) minutes are recommended. The same recommendations also count for older adults, permitted that their health status allows for it. Both the WHO and the AHA/ACSM recommendations do not explicitly mention the regularity of activity events, but refer to and use the ‘dose’ concept, related to the product of intensity (METs) and duration: MET minutes per week. However, the dose-effect relationship has been insufficiently researched with regard to the frequency distribution patterns, or in other words, regularity. Lee et al. report on the mortality risk of so-called ‘weekend warriors’ in the Harvard Alumni Health Study, defined as persons with an active energy expenditure of ≥1000 kcal/week during just one or two activity periods per week [9]. The regularly active (≥1000 kcal/week, >2 activity events) have a lower relative risk (0.64, 95% confidence interval: 0.55/0.73) than the weekend warriors (0.85, 95% CI: 0.65/1.11) in comparison to the insufficiently active (1.0, reference). This effect, however, was found significant only in men without cardiovascular risk factors among the weekend warriors, who have a relative risk of 0.41 (95% CI: 0.21/0.81). Within this group, men with at least one risk factor have a relative risk comparable to sedentary men (1.02, 95% CI: 0.75/1.38) [9]. Another definition of the weekend warrior pattern was given by Kruger et al. who characterized it as 150 minutes of moderate or vigorous physical activity per week (150 min/week), performed on one or two days [10]. The authors concluded that about 1–3% of U.S. adults belong to this activity group. Westerterp reported that high-intensity activity does not significantly predict overall physical activity levels, but moderate-intensity activities do in a study using the ‘Tracmor’ activity sensor device [11]. He concluded that moderate-intensity activities should be promoted, also because several subpopulations (older or obese persons) will not tolerate strenuous efforts.

Tudor-Locke et al. report on accelerometer data-based physical activity profiles in their analysis of the National Health and Nutrition Examination Survey (NHANES, n = 3522) [12]. The use a set of altogether 20 parameters describing volume (3), rate (3), duration (6), achievement of public health guidelines (1), transitions (1), and percentage spent at different activity levels (6). Their analyses are based on pre-processed data of an ActiGraph monitor, the parameters being ‘counts’ and ‘steps’. The aim of the analysis is to compare these parameters for three body mass index (BMI) groups, not to combine them to a new score or index [12]. The ‘rate’ parameters refer to steps or counts per minute and do not reflect the temporal distribution of activity events.

Metzger et al. also use NHANES accelerometer data (n = 3802) – with the three parameters daily minutes of moderate-to-vigorous activity and vigorous activity and minutes of moderate-to-vigorous activity in events lasting for at least 10 minutes – to identify different activity classes by using the technique of latent class analysis (LCA) [13]. Five classes are identified: two with low activity, one ‘weekend warrior’, one weekday active and weekend inactive and one active. The authors also included socio-demographic data for adjustment in their analysis (e.g. gender, education, age). Most (78.7%) of the participants are classified into the two low activity classes, and LCA produced no reliable results for the vigorous activity parameter.

Patnode et al. use LCA to determine gender-specific classes of activities in children [14]. However, the authors include parameters describing media (Internet, video games, TV, etc.) usage to determine and denote classes.

The author is not aware of any publications concerning a generic method to define different characteristic activity types from accelerometer data – including raw data – obtained in cohorts and including the relevant temporal distribution of activity episodes.

## Methods

The general approach is based on the assumption that a descriptive score which is useful to characterize activity patterns consists of the dimensions *duration*, *intensity* and *regularity* of activity events. Furthermore, the method should be adaptable to different cohorts such as older persons, athletes, or children. Next, data from different sensor devices or sources should be comparable with each other, as current cohort studies use different devices with divergent technical specifications. Finally, apart from mere numbers, a comprehensive graphical representation is necessary. All calculations to compute the ATLAS index – a three-dimensional vector – and the graphical plots have been performed using the *R Project for Statistical Computing* software environment (version 2.14.0, http://www.r-project.org/).

### Computation of ATLAS parameters: intensity, duration and regularity

Common methods to define activity intensity are based on METs or graduations in percentages of maximum oxygen uptake [6], [7], both of which are rarely available for participants in large cohort studies. On the one hand, there are only few devices able to measure METs, and on the other hand, exercise tests to measure maximum oxygen uptake reliably are time-consuming in a cohort protocol and potentially dangerous, and oxygen intake is difficult to measure or estimate over extended periods of time in an unsupervised environment. Therefore, a different measure which is derivable from raw (calibrated) accelerometer data and correlates with MET values, needs to be identified. In several publications, it has been proven that MET values and active energy expenditure are closely related to the signal magnitude area (SMA) calculated from accelerometer data, depending on the kind of activity a person performs [15]. Bouten et al. report a correlation of *r* = 0.95 under lab conditions [16]. The SMA is defined [15] as 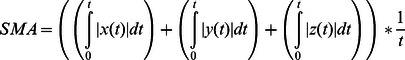



The SMA values were aggregated over periods of one minute and – to make comparison with MET values more comprehensive – increased by one. The latter was done because MET values by definition start with the value 1.

The second parameter – activity duration – was calculated by summing up all activities above a threshold intensity value, in this experiment a MET resp. SMA level of at least 1.5, in case an activity lasted longer than five consecutive minutes. This threshold was chosen for this experiment because long-term activity data of older persons from a study were used, and therefore high MET levels corresponding to, e.g., the AHA/ACSM recommendations for adults, could not be applied [7]. Also, five minutes were considered to be the minimum duration of an activity with considerate physiological resp. metabolic activation. Both thresholds, however, may be easily adapted to the specific characteristics of other cohorts if necessary. In order to make the duration sums comparable for data sets of diverging lengths, the author proposes to use the ratio of active minutes per 1000 recorded minutes.

The third parameter is regularity. As a fault-tolerant measure, the author proposes to use the variance of all time differences of detected activity periods lasting five minutes or longer. The activity events were marked in the aggregated data set, and then all differences were calculated. Because high variance values correspond to highly irregular activities and for reasons of clarity and comprehensiveness in graphical representation of the final results, the author proposes to use the following simple conversion: 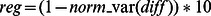 with *reg* as the regularity outcome measure and *norm_var* the normalized variance of all differences. If less than two events were detected and thus no difference was computable, *reg* was set to zero.

### Graphical representation

The graphical representation should allow for easy visual interpretation of the three-dimensional result vector and for identifying groups of persons belonging to distinguished activity pattern groups. A three-dimensional cube plot representation (function plot3d, R package *rgl*) was chosen. Based on physiological considerations and the literature sources mentioned above, five different activity types were chosen.

### Validation data sets

The author chose to evaluate the new method with 12 data sets, originating from three different long-term observational studies. Each patient has worn a wearable sensor for about one week. Table 1 shows the data set characteristics and the sources. Three different devices were used, with data in three stages – and thus deliberately covering the full range – of pre-processing: pre-processed and aggregated (*SenseWear Pro2*, Bodymedia, Pittsburgh, USA; LASS study: *Life*, *anxiety-free*, *self-sufficient and self-determined*), pre-processed calibrated (*ActiGraph GT3X*+, ActiGraph, Pensacola, USA; German National Cohort pre-study) and un-calibrated raw (*SHIMMER 2R*, Shimmer Research, Dublin, IRL; from a running outpatient fall risk study). The data sets are available for download via the website of the International Medical Informatics Association's (IMIA) working group ‘Wearable sensors in healthcare’ (www.wearable-sensors.org) or directly from the author.

**Table 1:** Sources of data sets used for the proof-of-concept study for the ATLAS index (*Activity Types from Long-term Accelerometric Sensor data*).

data set/patient #	cohort	device type	avg. duration per participant [days]	notes
1–9	LASS study [17]	SenseWear Pro2	5.4	device estimates METs
10	German National Cohort pre-study	ActiGraph GT3X+	7	calibrated raw accelerometer data
11–12	fall risk observation study	SHIMMER 2R	6.8	un-calibrated raw accelerometer data

The author is part of the teams conducting all three studies.

## Results

### Graphical reference model

**Figure 1:**
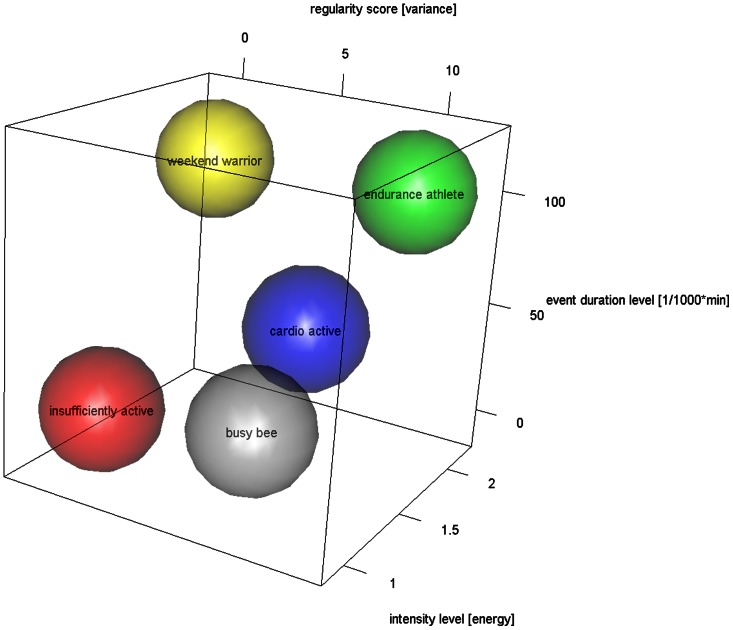
Proposed graphical representation of the ATLAS index as a three-dimensional cube. The three axes represent the regularity (x), the duration (y) in [mins/1000*mins] and the intensity (z). Five characteristic activity patterns are represented by coloured spheres.

Fig. 1 shows the proposed graphical representation as a three-dimensional cube. The three axes represent the regularity (x), the duration (y) in [mins/1000*mins] and the intensity (z). The author has identified five characteristic activity patterns which are represented by coloured spheres. Table 2 details the groups' characteristics. Catchy terms were chosen to figuratively represent the group's main characteristics, yet a common derogative term was avoided for the insufficiently active, because inactivity may be caused e.g. by age-related functional impairments or disease conditions and thus be unavoidable.

**Table 2:** Proposed activity type groups and value ranges for the three dimensions regularity, duration and intensity for graphical representation of the ATLAS index (*Activity Types from Long-term Accelerometric Sensor data*).

group name	regularity	duration	intensity
‘weekend warrior’	low	high	high
‘insufficiently active’	low	low	low
‘busy bee’	high	low	low
‘cardio active’	high	medium	medium
‘endurance athlete’	high	high	medium-high

### Proof-of-concept study

**Figure 2:**
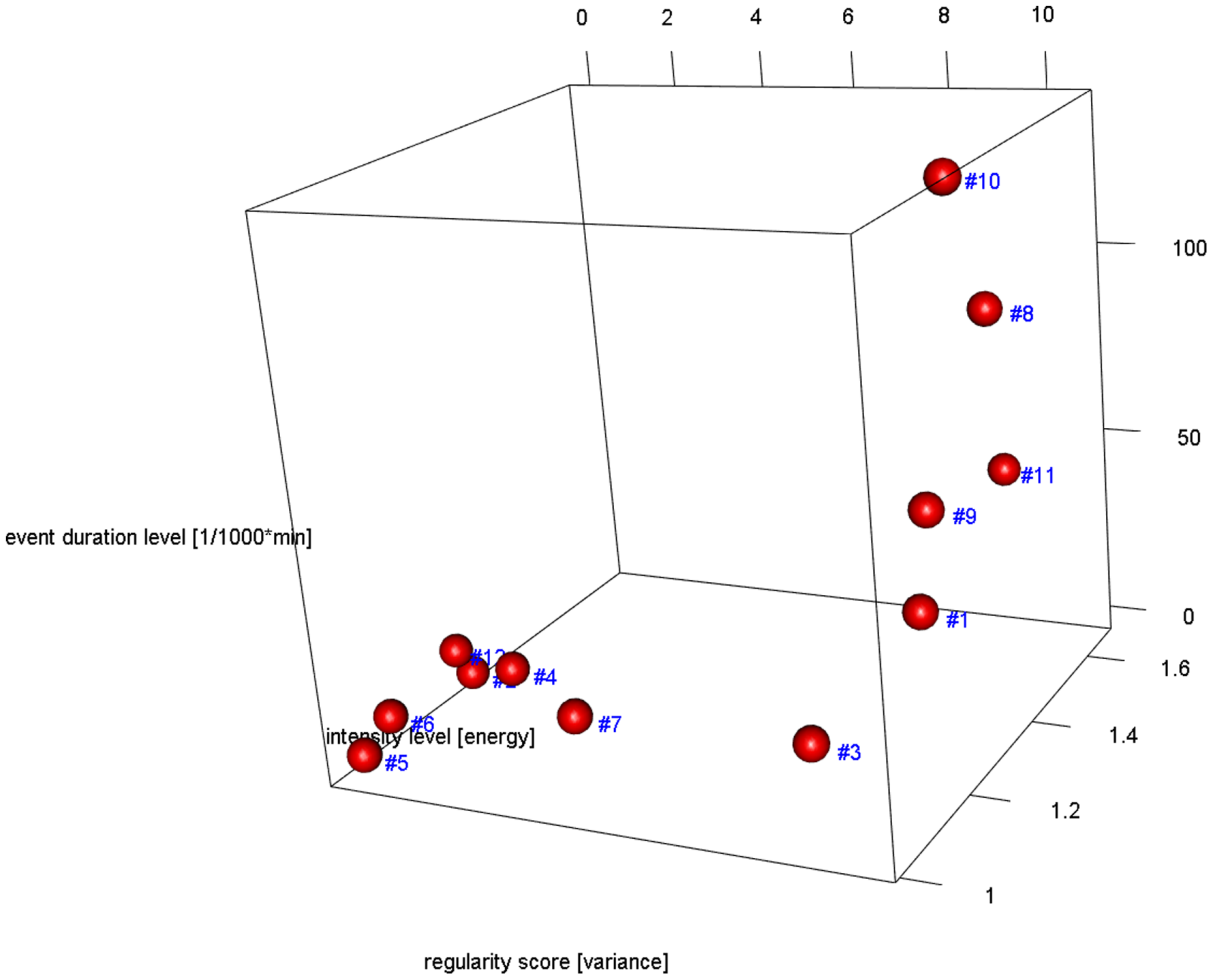
Three-dimensional representation of the 12 data sets used for the proof-of-concept study of the new ATLAS index. The three axes represent the regularity (x), the duration (y) in [mins/1000*mins] and the intensity (z).

Fig. 2 shows the 3D cube with all 12 data sets. Six data sets are located in the ‘insufficiently active’ area, whereas the other six data sets show higher regularity scores and are distributed along the duration axis. In the exemplary data sets, no ‘weekend warrior’ was present.

## Discussion

There is evidence that not only the duration and the intensity of physical activity are important to characterize the nature of physical activity, but also the regularity of activity events above a defined intensity and duration threshold. The elevated mortality risk of the weekend warrior at risk compared with a regularly active person reported by Lee et al. suggests that the temporal distribution plays an important role not to be disregarded when studying activity data in epidemiologic cohort studies [9]. Current scores using e.g. ‘MET-minutes’ [6], [10] cannot reflect this entity in the data and may therefore be insufficient to grasp the whole picture and thus may miss some of the information which lies dormant in the data sets. The ATLAS index accounts for temporal distribution by providing a simple measure of regularity and thus may be very useful for the many cohort studies interested in outcome measures related to activity and its patterns (aim #1).

The results show that data sets can be compared and distinguished using the ATLAS index, even if the devices used – and subsequently – the data sources are very different, ranging from raw un-calibrated accelerometer data to pre-processed and ready-made aggregated results obtained with device-specific undisclosed algorithms (aim #3). Thus, comparisons even between different cohorts are possible as the examples show. These inter-cohort comparisons, however, may only be qualitative because parameters computed with undisclosed algorithms (e.g., METs provided by the SenseWear Pro2 device) cannot be compared directly with the measures as suggested above. Within one cohort, however, distance measures between the three-dimensional vectors can be determined. The method is adaptable to different groups or sub-cohorts such as older persons, children or athletes, and allows for activity type comparisons within and among them. It provides an abstracted and simple vector score that is comprehensive and easily represented in graphical form.

The suggested graphical representation using a three-dimensional cube with the axes regularity (x), duration (y) and intensity (z) makes the characteristics of the data sets easily accessible, and single data points or clusters can be compared visually (aim #2). The five archetypical groups proposed are based both on literature and common physiological domain knowledge. These groups are thus far a mere proposal and have to be confirmed and probably adapted with regard to their position and extent. While the 12 exemplary data sets may be assigned by approximation to four of these groups (insufficiently active: #2, #4, #5, #6, #7 and #12; busy bee: #1 and #3; cardio active: #8, #9 and #11; endurance athlete: #10), no weekend warrior type was present in our study. This result is expected as only about 1–3% of adults belong to this group [10] and because only one participant was aged below 50 years (#10), the other data sets coming from older persons resp. patients with a high fall risk.

### Limitations

Neither the proposed groups nor the ATLAS index have been validated so far with a large number of data sets, so a validation to confirm the groups and to find their reference centroids has to be conducted. In addition to this approach based on domain knowledge, a machine-learning method could be used to identify different activity groups or confirm the proposed ones, e.g. using clustering or latent class analysis [13], [14]. Furthermore, so far the proposed regularity parameter does not account for diurnal variations, which might add more information. Additionally, the intensity parameter currently does not account for individual factors influencing active energy expenditure, such as body weight. Such factors, however, may easily be included in the computation. Finally, the duration threshold of five minutes for relevant activity periods is to some extent arbitrary and differs from the 10-minute threshold used e.g. by Metzger et al [13]. Nevertheless, the threshold is easily adaptable but has been chosen deliberately to account for the fact that the author frequently analyzes data from cohort studies with older or functionally impaired persons which are not able to perform long-term high-intensity activities.

## Conclusions

This paper presents a new index to characterize physical activity patterns on the basis of long-term accelerometer data recorded using wearable devices. The ATLAS index (Activity Types from Long-term Accelerometric Sensor data) is a three-dimensional vector consisting of the parameters duration, intensity and regularity, the latter being little regarded so far yet potentially relevant. Along with the new index a 3D cube graphical representation to clearly display the results is proposed. The feasibility of the approach is demonstrated with exemplary accelerometer data sets from three different devices and cohorts. Further research work has to be conducted to validate the new index using a large data set and to confirm the five proposed archetypical activity types.
